# Developing an Assessment Tool of Nursing Home-Based Advance Care Planning Implementation Quality

**DOI:** 10.1093/geroni/igaf122.154

**Published:** 2025-12-31

**Authors:** Peiyuan Zhang, Joan Davitt, Nancy Kusmaul, Paul Sacco, Kathleen Unroe, Kaipeng Wang, Fei Sun, John Cagle

**Affiliations:** University of Maryland, Baltimore County, Baltimore, Maryland, United States; University of Maryland, Baltimore County, Baltimore, Maryland, United States; University of Maryland, Baltimore County, Baltimore, Maryland, United States; University of Maryland, Baltimore, Maryland, United States; Indiana University School of Medicine, Indianapolis, Indiana, United States; University of Denver, Denver, Colorado, United States; Michigan State University, East Lansing, Michigan, United States; University of Maryland, Batimore, Maryland, United States

## Abstract

Advance care planning (ACP), a key mechanism to improve the quality of end-of-life care, has significant relevance in nursing homes (NHs) where nearly 30% of older Americans spend their last days, but the quality of ACP practices vary. Thus, this study aimed to develop a tool measuring quality of NH-based ACP implementation. Firstly, we conducted a systematic review of quality indicators and factors influencing NH-based ACP implementation. Guided by Donabedian’s model of quality of care, we conducted deductive thematic analysis of the selected 19 studies and identified 13 elements for successful ACP implementation. They were categorized into two major dimensions: (1) nursing home structural support for ACP and (2) standardized implementation procedures and converted into binary questions to form the drafted tool. Then a Delphi study was conducted with 18 clinical experts with an average of 23 years working with NH residents to revise the tool. Almost half of participants conducted more than 10 ACP conversations in a typical month. The first round involved 5 focus groups on their opinions about the drafted tool, resulting in the modified tool including 19 questions. It was presented back to panelists in Delphi surveys. Response options were 1=endorse, 2=would endorse with modification, and 3=not endorse. The consensus was reached after two rounds of rating (interquartile range=0, median=1, percentage of agreement>80%). To our knowledge, this is first tool examining ACP implementation quality at the NH level, which has the potential to guide ACP policy and interventions to facilitate standardized procedures and enhance NH buy-in.

